# Notes

**Published:** 1899-07-22

**Authors:** 


### NOTES.

The Royal Hospital for Women and Children is to be rebuilt. The cost is estimated at £30,000.

The new premises of the Surgical Aid Society have been opened by the President, the Earl of Aberdeen.

In view of the increased number of patients suffering from infectious diseases remaining under treatment in the hospitals of the Metropolitan Asylums Board, and of a report presented by the Ambulance Committee on the subject of the probable requirements of London in the near future, the Managers have decided to open their new hospital—the Grove Hospital—at as early a date as possible. Dr. Caiger, from the South-Western Hospital, will be appointed medical superintendent; and Miss Wacher, from the Small-pox Ships, will be matron.

In answer to a question by Sir W. Foster in the House of Commons, Mr. Brodrick said: "Her Majesty's Government believe that the principles of the Venice (Plague) Convention of 1897 are sufficient to protect any country adopting them from the danger of an invasion of plague. That Convention is based on the principle that a country should place its sanitary organisation in such a condition that it can easily deal with any case of plague that may be imported into or be detected in its territory." Rather a snub for the sanitary administration of India, where the plague has had the upper hand for the past three years, and for Hong-Kong, a Crown colony, where it has repeatedly recurred!

All the wards at the Great Northern Central Hospital are now open. The last remaining ward to remain closed was opened in consequence of a grant from the Prince of Wales's Fund, which was made conditionally for this purpose.

In addition to the ophthalmic school to be erected at Brentwood for 360 children, the Metropolitan Asylums Board have sanctioned the plans for another similar school at Swanley. The architects (Messrs Newman and Newman) estimate the cost of the building at £110,524, an amount somewhat higher than the estimate (£106,600) for the schools at Brentwood.

It is evident that public opinion is moving in the right direction when both Houses of Parliament concern themselves with the question of seats for shop assistants. A Bill for Scotland was before Parliament some time since, but it was rejected on the ground that England and Ireland were excluded. The present Bill was for England and Ireland, but Scotland has now been included, and the Bill providing one seat for every three female shop assistants is before the House of Lords. It seems very strange that legislature should have to be resorted to on this subject, seeing that it would have been very easy for a few influential ladies to combine and insist upon seats being provided in shops they patronised, making their custom contingent upon the provision. It is not very creditable to the women of England to allow it to be necessary that so simple a matter for the welfare of their sex should be remedied by male legislators. This is a time when women are agitating for more power in affairs, yet they permit so obvious an opportunity to assist those of their own sex to pass out of their hands. If the provision of seats for shop assistants becomes a matter for the State further expense will be put upon the ratepayer, as to enforce the law inspectors will have to be provided.

